# Multi-Layer Preprocessing and U-Net with Residual Attention Block for Retinal Blood Vessel Segmentation

**DOI:** 10.3390/diagnostics13213364

**Published:** 2023-11-01

**Authors:** Ahmed Alsayat, Mahmoud Elmezain, Saad Alanazi, Meshrif Alruily, Ayman Mohamed Mostafa, Wael Said

**Affiliations:** 1 Department of Computer Science, College of Computer and Information Sciences, Jouf University, Sakaka 72341, Saudi Arabia; sanazi@ju.edu.sa (S.A.); mfalruily@ju.edu.sa (M.A.); 2 Computer Science Division, Faculty of Science, Tanta University, Tanta 31527, Egypt; mmahmoudelmezain@taibahu.edu.sa; 3 Computer Science Department, College of Computer Science and Engineering, Taibah University, Yanbu 966144, Saudi Arabia; 4 Information Systems Department, College of Computer and Information Sciences, Jouf University, Sakaka 72341, Saudi Arabia; 5 Computer Science Department, Faculty of Computers and Informatics, Zagazig University, Zagazig 44511, Egypt; wmohamed@taibahu.edu.sa; 6 Computer Science Department, College of Computer Science and Engineering, Taibah University, Medina 42353, Saudi Arabia

**Keywords:** retinal image, noise removal, data imputation, data augmentation, GAN, segmentation

## Abstract

Retinal blood vessel segmentation is a valuable tool for clinicians to diagnose conditions such as atherosclerosis, glaucoma, and age-related macular degeneration. This paper presents a new framework for segmenting blood vessels in retinal images. The framework has two stages: a multi-layer preprocessing stage and a subsequent segmentation stage employing a U-Net with a multi-residual attention block. The multi-layer preprocessing stage has three steps. The first step is noise reduction, employing a U-shaped convolutional neural network with matrix factorization (CNN with MF) and detailed U-shaped U-Net (D_U-Net) to minimize image noise, culminating in the selection of the most suitable image based on the PSNR and SSIM values. The second step is dynamic data imputation, utilizing multiple models for the purpose of filling in missing data. The third step is data augmentation through the utilization of a latent diffusion model (LDM) to expand the training dataset size. The second stage of the framework is segmentation, where the U-Nets with a multi-residual attention block are used to segment the retinal images after they have been preprocessed and noise has been removed. The experiments show that the framework is effective at segmenting retinal blood vessels. It achieved Dice scores of 95.32, accuracy of 93.56, precision of 95.68, and recall of 95.45. It also achieved efficient results in removing noise using CNN with matrix factorization (MF) and D-U-NET according to values of PSNR and SSIM for (0.1, 0.25, 0.5, and 0.75) levels of noise. The LDM achieved an inception score of 13.6 and an FID of 46.2 in the augmentation step.

## 1. Introduction

Segmentation is one of the most significant tasks in the field of computer vision and image processing, especially in the medical field. Medical segmentation is the process of splitting or identifying certain structures or regions of interest within medical pictures. Each region depicts an area with similar features, which can include issues such as color, density, texture, or other visual attributes. The segmentation process helps many physicians diagnose and examine many diseases. Recently, deep learning (DL) has been involved in the process of segmenting numerous medical images of the brain, breast, heart, and blood vessels [1,2,3,4,5]. It is worth noting that DL has proven particularly valuable in segmenting blood vessels in the retina, helping ophthalmologists and medical professionals in early detection of various eye and systemic diseases [6,7]. The retinal vascular system, also known as the retinal vasculature, is a network of blood vessels located within the eye’s retina. Besides the importance of the retina for vision, retinal vascular changes are often early indicators of various ocular diseases and human body diseases as a whole. Ocular diseases include retinal artery occlusion, retinal vein occlusion, and retinal vein occlusion. Human body diseases include diabetic retinopathy, hypertensive retinopathy, macular degeneration, systemic inflammatory conditions, atherosclerosis, and hematological disorders. Indeed, regular monitoring of the retinal vasculature can help in the early detection of such diseases. Therefore, accurate and automated retinal vessel segmentation is crucial for early diagnosis, monitoring, and detection of these diseases, helping ophthalmologists and medical practitioners make more informed clinical choices [8,9,10,11]. There are numerous image segmentation techniques, each with its own advantages, drawbacks, features, applications, and use cases [12,13]. These methods can be classified as either conventional image segmentation techniques or methods based on deep learning. Conventional image segmentation approaches encompass threshold, region-based analysis, edge-based techniques, watershed methods, and clustering-based methods. Recently, DL presented many models for segmenting retinal fundus images, such as convolutional neural networks (CNN), fully convolutional networks (FCN), and encoder–decoder-based models, i.e., U-Net [14,15,16]. The U-Net and its variant architectures, such as U-Net++ and residual U-Net, prove their efficiency when compared with other DL models because of their accuracy and a small number of parameters during the training process [17,18]. Preprocessing of retinal images is a highly significant task before segmentation for increasing the accuracy of the segmentation and training process. Preprocessing comes in various forms, including the elimination of diverse image noise, the augmentation of datasets, and the imputation of missing data [19,20,21]. The primary goal of this research is to highlight the role of preprocessing in influencing the segmentation results of fundus images. Specifically, the focus is based on data imputation, noise removal, and image augmentation. 

Noise in medical images is undesired change in pixel densities or values that can significantly affect the quality of images. This, in turn, can lead to negative consequences during the training process, affecting the final accuracy of segmentation. As a result, the accuracy of diagnosis and treatment planning for patients can be affected. Noise can be introduced at different stages of the imaging process, from image acquisition to transmission and storage [22,23]. Various types of noise can have a negative impact on medical images, including salt-and-pepper, speckle, and amplifier noise. Many methods can be employed to reduce noise, ranging from traditional techniques such as Gaussian and mean filters to modern methods such as machine learning (ML)-based methods, as well as deep DL-based methods such as auto-encoders and generative adversarial networks (GANs). The effectiveness and efficiency of DL in removing and decreasing noise in medical images has been validated, particularly in the case of images representing ocular blood vessels, i.e., retinal fundus images [24]. Removing noise from retinal images is one of the most significant components in the proposed multi-layer preprocessing approach.

There is a direct relationship between the segmentation process performance and the number of elements in a dataset. Enlarging or expanding small datasets effectively enhances the segmentation process’s accuracy. Data augmentation is a technique to artificially expand the training set by generating modified versions of a dataset using existing data [25]. In the literature, there are many generative DL models, such as GANs, variation auto-encoders (VAEs), and diffusion models, that have been used in generating images [26,27]. Nonetheless, these generative models have drawbacks when used to create high-quality samples from challenging, high-resolution datasets. For instance, VAE models frequently have sluggish synthesis speeds, whereas GANs frequently experience unstable training and mode collapse [28]. The latent diffusion model (LDM), a class of generative diffusion models, has received significant attention recently in the field of data augmentation [29]. In this paper, the LDM is employed to generate synthetic retinal fundus images as another step in the proposed multi-layer preprocessing approach.

Data imputation is another critical component of the proposed multi-layer preprocessing approach; its effectiveness can significantly impact the results of the segmentation process. The main purpose of data imputation is to properly handle missing data by generating reliable approximations of missing values. This may be accomplished using numerous imputation methods, which can range from simple techniques like mean imputation to more complicated approaches like DL-based techniques [30]. DL-based medical image imputation has gained great importance due to the remarkable capabilities of DL models in capturing complex patterns and structures in medical images. In this paper, DL-based image imputation techniques are used to reconstruct missing data in retinal fundus images to increase the performance of the retinal blood vessel segmentation process.

Preprocessing is an indispensable step in the context of retinal blood vessel segmentation using fundus images. It plays an essential role in improving image quality and facilitating the accuracy of the segmentation process. In this paper, a multi-layer preprocessing approach comprising three distinct layers is proposed. The first layer is used to reduce noise sources, resulting in sharper images for segmentation. The second layer is to utilize dynamic data imputation techniques for estimating missing vessel segments to enable more comprehensive vessel network analysis. The third layer increases the size and diversity of the dataset using an LDM model to enhance the robustness and generalizability of the segmentation process. The following is a concise outline of the paper’s contributions:

1. Introduces a novel framework that pioneers a multi-layer preprocessing approach, consisting of three stages: noise reduction, dynamic data imputation, and data augmentation. This comprehensive preprocessing strategy provides a holistic solution to the complexities associated with retinal image data, enhancing the quality of input for subsequent segmentation.
2. The framework significantly boosts segmentation performance, resulting in impressive accuracy and precision in the segmentation of retinal blood vessels. The utilization of the U-Net with a multi-residual attention block (MRA-UNet) for this purpose underscores the framework’s effectiveness in this critical task.
3. Demonstrates the framework’s versatility by effectively addressing challenges such as noisy images, limited datasets, and missing data. The proposed methods in noise reduction, data imputation, and data augmentation collectively contribute to the framework’s adaptability to various real-world scenarios.
4. The framework exhibits remarkable efficiency in noise removal, as evidenced by the values of PSNR and SSIM for different noise levels. The application of the CNN with matrix factorization (MF) and D-U-NET methods for noise reduction reinforces its capability in enhancing image quality.
5. The LDM plays a vital role in augmenting the training dataset, contributing to the model’s success.

## 2. Related Work

Research has shown that retinal blood vessel shape is associated with metabolic risk and other disorders. As the eye is a sensory organ, every eye condition significantly impacts how the brain processes sensory information and draws conclusions. One of the serious eye conditions for which a novel treatment is needed is choroid neovascularization. The choroid is where blood vessels develop. Many scientific research projects have introduced DL models for segmenting the retinal blood vessels, such as convolutional neural network (CNN), artificial neural network (ANN), auto-encoders (AEs), fully convolutional networks (FCN), and U-Net [31,32]. During the analysis of medical images, the U-Net design is considered a great and powerful architecture, especially in relation to retinal vascular segmentation. It promises to improve early disease detection, treatment monitoring, and general care for patients in the field of ophthalmology [33] because it is highly effective at precisely recognizing blood vessels in retinal images. The segmentation of retinal blood vessels using various U-Net designs is explored in this study, given the prevailing adoption of this technology and it having achieved significant accuracy and reliability.

As presented in [34], the authors proposed the U-Net architecture as a complete convolutional neural network (FCN) applied for the segmentation of biomedical images. It comprises an encoder, decoder, and skip connections organized in a U-shaped configuration. Indeed, the well-known use of the U-Net architecture in the biomedical field and its significant impact on medical image segmentation cannot be denied. The U-Net framework is employed in the segmentation of medical images, including tasks like brain tumor segmentation, cardiac image segmentation, skin lesion segmentation, and retinal blood vessel segmentation, as demonstrated in previous studies [17,35,36]. 

The authors of [37] provided an improved version of the U-Net model to segment retinal blood vessels. The conventional U-Net is given a multiscale input layer and dense blocks so that the network can utilize more detailed spatial context data. The DRIVE public dataset tests the authors’ suggested technique, which received scores of 0.8199 for sensitivity and 0.9561 for accuracy. The results of segmentation have improved, particularly for small blood vessels that are challenging to identify due to their low pixel contrast. 

As shown in [38], a U-Net attention mechanism is presented for retinal vessel segmentation. The channel and location attention modules are both parts of the attention mechanism. The channel attention module constructs the feature map’s many channels’ long-range dependencies. The feature map’s regions’ long-range relationships are constructed using the position attention module. Images are divided into 250 × 250 pixel patches for preprocessing, and the patches are then rotated and flipped. The DRIVE dataset is used to assess the proposed model. The dice entropy loss function, a new loss function for the data imbalance problem, lets the model concentrate more on the vessel.

Gargari et al. [39] presented a multi-stage framework for fundus image segmentation and eye-related disease type diagnosis. The retinal blood vessel segmentation process is conducted using the U-Net++ model for the green channel of fundus images. While the eye-related diseases are diagnosed using CNN. Preprocessing stages are utilized before the segmentation process. The preprocessing stages include improving the quality of images using the histogram normalization method, removing noise using the Gaussian filter, and applying the Gabor filter. Following the segmentation process, the subsequent phase involves the extraction of HOG and LBP features for disease diagnosis. The effectiveness of the suggested framework is assessed using the DRIVE and MESSIDOR datasets. Although the proposed multi-stage framework achieved significant results, the impact of the preprocessing stages is not clearly known.

A residual attention UNet++ (RA-UNet++) for medical image segmentation is described in [40]. By including a residual unit with an attention mechanism, it improves the U-Net++ model. As a result, the degrading issue is recovered by the residual unit. The significance of the background areas that are unrelated to the segmentation task is diminished while the significance of the target region is increased by the attention process.

In [41], a U-Net 3+ model is introduced, which is essentially a U-Net with full-scale skip connections and deep supervision, tailored for segmentation of medical images. These skip connections seamlessly blend intricate details with significant semantic information gathered from feature maps of varying scales. These comprehensively amalgamated feature maps are then leveraged by the deep supervision technique to facilitate the training of hierarchical representations. More recently, Xu et al. [42] enhanced the U-Net 3+ model by streamlining the full-scale skip connections and incorporating an attention-based convolutional block module to collect crucial features. The efficacy of this model was substantiated through evaluations in tasks encompassing the segmentation of skin cancer, breast cancer, and lung cancer.

The authors of [43] introduced the spatial attention U-Net (SA-UNet) as a lightweight model designed for blood vessel segmentation. The core concept behind the SA-U-Net is to replace the U-Net’s convolutional block with a structured dropout convolutional block that combines both Drop_Block and batch normalization to prevent the network from overfitting. Additionally, the SA-U-Net incorporates a spatial attention module, which serves to emphasize important features while suppressing less crucial ones, thereby enhancing the network’s capacity to effectively represent data. Prior to the segmentation process, various data augmentation techniques are applied. These techniques encompass random rotation, the introduction of Gaussian noise, and color adjustment, as well as horizontal, vertical, and diagonal flips. The evaluation of this model is carried out using the DRIVE and CHASE DB1 datasets.

The authors of [44] proposed a new deep learning model called DEU-Net, which is specifically designed for segmenting retinal blood vessels. DEU-Net uses an end-to-end pixel-to-pixel approach, meaning that it takes an image as input and produces a segmentation mask as output in a single step. DEU-Net has two encoders, one for preserving spatial information and the other for capturing semantic content. The spatial encoder extracts features that represent the location of pixels in the image, while the semantic encoder extracts features that represent the meaning of pixels. DEU-Net also uses a channel attention mechanism to select the most important features from each encoder. This helps to improve the accuracy of the segmentation results.

A deep learning network called Vessel-Net is intended to precisely segment retinal blood vessels. It is a condensed model that improves feature representation by fusing the benefits of the residual module and the inception model. Four distinct supervision paths are included in Vessel-Net’s multi-path supervision technique, which aims to guarantee that the model learns rich and multi-scale characteristics. In addition, a preprocessing step is used by Vessel-Net to lower noise and boost contrast in the input photos. Vessel-Net demonstrated state-of-the-art performance on both of the public retinal image datasets, DRIVE and CHASE, where it was tested [45]. 

In order to enhance feature representation, a number of studies have suggested modifying the U-Net model for retinal blood vessel segmentation by adding residual attention blocks. The RA-UNet was proposed by Ni et al. [46], Zhao et al. Dong et al.’s attention_res UNet was proposed in [47]. Guo et al. proposed the CRA U-Net in [48]. The channel attention residual U-Net was proposed by [49], and Yang et al. A residual attention model with dual supervision was put forth by [50]. Using a multi-residual attention block (MBA), a densely connected residual network with an extra attention mechanism, we developed the MRA-UNet in our own research.

Although many architectures have been introduced for segmenting the retina’s blood vessels based on U-Net, all of these architectures have some advantages and have efficient accuracy. However, they cannot deal with small datasets and noisy images. As presented in Table 1, different architectures of the U-Net are provided to explain the main characteristics of the blood segmentation of the retinal vessels. The table explains the main advantages and disadvantages of the DL model.

## 3. Methodology

**Figure 1:**
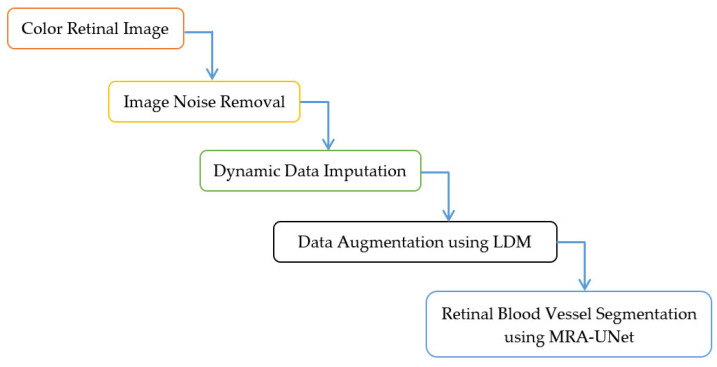
Framework for the proposed methodology.

This section presents the methodology for the retinal blood vessel segmentation framework, which encompasses two stages. It starts with the preprocessing stage and ends with the segmentation process stage using U-Net with multi-residual attention block (MRA-UNet). The preprocessing stage contains three layers namely, removing noise from retinal fundus images, dynamic data imputation, and data augmentation using LDM. Figure 1 and Algorithm 1 indicate the steps of the proposed framework. In Section 3.1, the DRIVE dataset, which contains retinal fundus images, is described. In Section 3.2, The noise elimination layer is explored. In Section 3.3, the dynamic data imputation layer is discussed. Section 3.4 is devoted to presenting the data augmentation layer. The retinal blood vessel segmentation process is indicated in Section 3.5. In Section 3.6, the utilized hardware and software specifications are tabulated. Section 3.7 is dedicated to the discussion of the diverse evaluation metrics used in this study. 

**Algorithm 1:** Data Augmentation and Segmentation1**Input** ← Retinal Image Dataset2  **Initialize Preprocessing Stage**
3  **Step 1:** Noise Removal4   Apply a U-shaped CNN with Matrix Factorization5   Reduce Image Noise6   Apply D-U-Net to reduce image noise7   Choose best Free_Noise_Image using PSNR and SSIM8  **Step 2:** Dynamic Data Imputation9   Apply Multiple Imputation Models10   Fill Missing Data in Retinal_Image11   Generate Imputed Retinal_Image12  **Step 3:** Data Augmentation13   Apply LDM to augment training dataset14   **FOR** EACH Retinal_Image **DO**15    Generate Multiple Augmented Images using LDM16   **END FOR**
17  **Initialize Segmentation Stage**
18  Apply U-Net with a multi-residual attention block (MRA-UNet)19  Segment Preprocessed & Free_Noise_Image20  **INSERT** Preprocessed & Free_Noise_Image **INTO** U-Net21**Output** → Segmented Retinal Image

### 3.1. DRIVE Dataset

**Figure 2:**
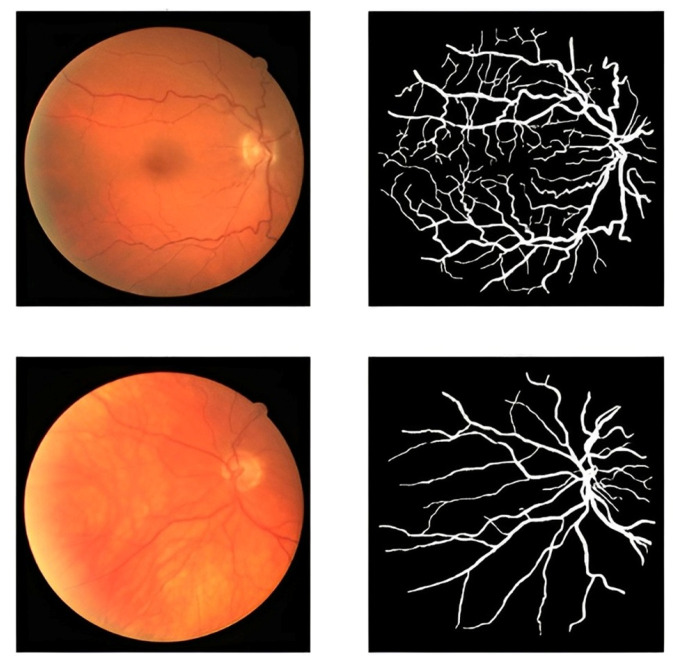
Blood vessel of retinal images and masks.

The proposed framework in this study uses an accessible dataset called the DRIVE dataset [51]. The dataset contains 40 retinal images. They were obtained at a resolution of 768 × 584 pixels with 8 bits per color plane. A number of 33 images do not exhibit any evidence of diabetic retinopathy, while 7 images have early moderate indicators of the disease. Several retinal images and blood vessels from the DRIVE dataset are shown in Figure 2. The number of these images is so limited for an efficient segmentation process. To address the limited size of the dataset and enhance its diversity, we employed data augmentation techniques.

### 3.2. Removing Noise

This section presents two distinct models used to remove noise in retinal images. The choice of the most appropriate model is determined based on the PSNR value and noise level. In Section 3.2.1, the utilization of U-shaped CNN with matrix factorization is introduced. In Section 3.2.2, the application of denoising U-shaped Net (D-U-Net) model is outlined.

#### 3.2.1. Removing Noise Using U-Shaped CNN with Matrix Factorization

Li [52] presented multi-stage progressive CNN with a matrix factorization block framework for removing noise from images. The framework is composed of a dual-stage horizontal U-shaped structure to address the challenge of global structured feature extraction. The author proposed an improvement to the U-Net by introducing a matrix factorization denoising module (MD), a cross-stage feature fusion module (CSFF), and a feature fusion module (FFU). The matrix factorization (MF) method effectively fills gaps during de-noising. The architecture of the model contains three parts: (a) the de-noising module (MD), (b) the coder block, and (c) feature fusion module (FFU). The MD simulates the interplay between obtaining context information and aggregating global context. To enhance the flow of information and maintain network efficiency, the model redesigns a fundamental building block. The FFU based its decisions on data from several sources. 

**Figure 3:**
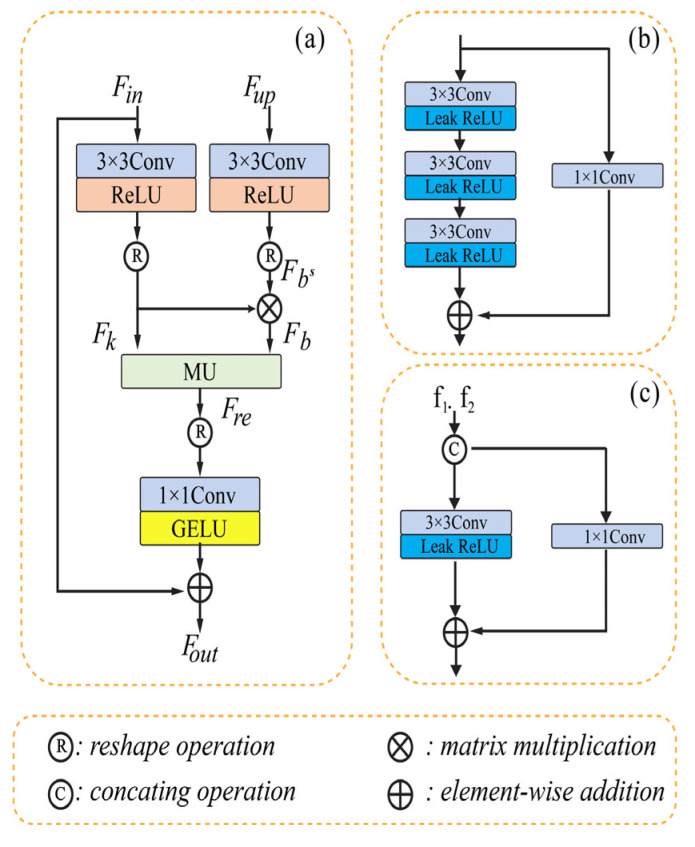
Removing noise using U-shaped CNN with MD [52]. (**a**) represents the MD module. (**b**) represents the Coder block. (**c**) represents the e FFU module.

In order to gradually rebuild the de-noised image, we employ two-stage convolution branches and draw inspiration from the design of multiple-stage progressing regeneration. Low-level computer vision tasks sometimes overlook the importance of the detail characteristic in recovering the image, which instead directly stack the convolution layer to identify the features. The leak Relu has a fixed slope of 0.02 and the 3 × 3 convolution layer comprises the coder’s unit. It consists of shortcutting using the 1 × 1 convolution and stacking three units. The model’s MD section contains three convolution layers (3 × 3) with the leak Relu function, which are then added to another convolution layer (1 × 1). The third part contains only one convolution layer of size 3 × 3 and uses element-wise addition as in the previous module. The FFU module exchanges and integrates data from various channels before the MD module, the decoder, and between two succeeding stages. The input matrix is factored into two submatrices by the MD module, which then reconstructs the matrix to provide the structured feature. The multiplicative updating procedures are then used. Figure 3 shows the typical architecture of the three different modules of U-shaped CNN with matrix factorization.

#### 3.2.2. Removing Noise Using D-U-NET

The denoising U-shaped Net (D-U-Net) [53] is utilized to remove speckle noise from retinal images. The D-U-Net model is structured into two components: the contraction and the expansion components. The contraction component incorporates a ‘max pool layer’ to downsize the initially generated image as a preprocessing step before the denoising process. The expansion component restores the image to its original dimensions after noise removal from the generated images by utilizing transpose convolution layers instead of the up-sampling layer. The D-U-Net architecture was trained using an Adamx optimizer; the learning rate was set to 0.0001, and the training was conducted with batch sizes of 128 and over the course of 100 epochs. The model employs the factorization module to reconstruct missing data and fill gaps during the restoration process after noise removal.

### 3.3. Dynamic Data Imputation

Data imputation can help estimate the missing vessel segments in fundus images. Different data imputation models are used to estimate missing vessel segments. These models include the multivariate imputation by chained equations (MICE) [54], GAIN [55], auto-encoder (AE) [56], L2 regularized regression (L2RR) [57], reinforcement learning- based approach (RL) [58], Neural Network Gaussian Process (NNGP) [59], probabilistic nearest-neighbor (PNN) [60], and modified GAIN [61]. The best model is selected according to the error value of the root mean square (RMSE) and Freshet Inception Distance (FID). The dynamic data imputation method [62] is applied by obtaining new imputed values at each training epoch. 

The modified GAIN is a Wasserstein GAN with an identity block. The identity block is important in the context of Wasserstein GAN as it ensures the preservation of original features, improves the accuracy of gain estimation, and enhances the stability of the training process. By incorporating the identity block, generative models can achieve more reliable and robust performance in data imputation, leading to better quality and more faithful representations of the real data distribution.

The modified GAIN’s basic principle is to employ deconvolution in both the generator and discriminator. To overlapping regions of the data that have been shifted around, convolution provides a kernel. Convolutional kernels are actually relearning old data because of the strong correlations in the actual data. The training of neural networks is difficult because of this redundancy. Before the data is passed into each layer, the deconvolution can eliminate the correlations.

All the models are trained using 200 epochs, an Adamx optimizer, and a 0.0001 learning rate. When using real data vectors in GAIN, the generator component G fills in the values that can be missing based on the identified observed data. The discriminator component D then acquires a finished vector and distinguishes between the observed and synthesized elements. A hint vector is used as supplementary information for discriminator D to identify the required dissemination in the component G. By utilizing the concept of network deconvolution, we enhance the GAIN models. 

Because many image-based datasets have substantial correlations, convolutional kernels typically relearn duplicated data. Although the deconvolution technique has been successfully used on images, the GAIN method has yet to be subjected to it. The model has a batch normalization vector and a linear layer. Preventing training problems like disappearing or exploding gradients, adjusting inputs to a mean of zero and the unit variance, using an up-sample layer and a convolution layer to learn from the up-sample layer, and using Relu for the generator all contribute to stabilizing learning. 

### 3.4. Data Augmentation Using LDM

In this layer, the LDM is utilized for data augmentation. The LMD integrates the computational properties of diffusion models with the use of auto-encoders, to compress the input data into a lower-dimensional latent representation. The auto-encoder was trained using L1 loss as well as perceptual loss. L1 loss, perceptual loss, a patch-based adversarial goal, and a structure of the latent space were used to train the auto-encoder.

The retinal fundus image is converted by the encoder into a latent representation with (20 × 28 × 20) dimensions. The latent data from the training set are input into the diffusion framework once the compression framework has been trained. LDM employs an iterative de-noising procedure to transform Gaussian noise into samples from a learned data distribution. Using a fixed Markov chain with 1000 iterations and a latent illustration of an example from our training set, the diffusion algorithm gradually obliterates the data structure while introducing Gaussian noise in accordance with a predetermined linear variance schedule.

### 3.5. Residual Attention U-Net Segmentation

The MRA-UNet is a customized U-Net model designed for accurate retinal blood vessel segmentation. It closely resembles the residual attention U-Net, but with the key difference of multi-residual blocks. The MRA-UNet architecture consists of an encoder and decoder, with skip-connections that combine features at different scales. The multi-residual blocks modify the initial convolutional layers and increase the depth of the network.

**Figure 4:**
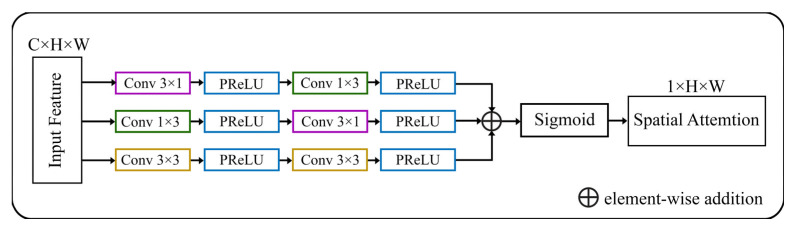
Architecture of spatial augmented attention module [63].

A spatial augmented attention module is utilized from [63] as an enhanced attention module. The spatial attention module is incorporated as a residual attention block. This block takes the feature map from the encoder part of the U-Net and applies attention to selectively highlight important spatial locations or regions. Because low-level qualities lack semantic significance, the spatial attention block supplies crucial background information. This data may complicate the segmentation process for the target item. Figure 4 shows an attention block in the MRA-UNet model. 

The enhanced attention module was introduced to accept high-level semantic data and accentuate target elements to solve the mentioned issue. The location is gained by the decoder using up-sampling. Nevertheless, this results in the loss of location data and the blurring of edges. The skip connections are used to mix low-level characteristics with high-level features. Because low-level traits lack semantic significance, they supply superfluous background information, which may need to be clarified by the segmentation of the target item. The enhanced attention module was designed to extract high-level semantic information and highlight target elements to address this issue. The MRA-UNet model and all other models are trained across 200 epochs with a learning rate of 0.0001 and 256 batch sizes. 

By incorporating the spatial attention mechanism as a residual attention block, MRA-UNet can effectively capture spatial dependencies and adaptively attend to relevant regions during the segmentation process. This helps improve the model’s segmentation performance by enhancing the representation of important features and suppressing noise or irrelevant information.

### 3.6. Hardware and Software Specification

Table 2 shows the hardware and software specifications that have been used during the training process in both augmentation and segmentation experiments.

### 3.7. Metrics Evaluation

Evaluating the quality and diversity of generated images is a crucial aspect in the evaluation of generative models. Two commonly utilized metrics for this purpose are *IS* (inception score) and *FID* (Fréchet Inception Distance). These metrics offer quantitative measures to evaluate the performance of generative models in terms of image quality and diversity. The inception score metric utilizes a pre-trained inception model, typically trained on a comprehensive dataset like ImageNet. It evaluates the quality of generated images based on two primary criteria: image quality and diversity. The calculation equation for the inception score is as follows:

(1) $$IS=exp\left(E_{x\sim p_{g}}D_{KL}\left(p\left(y|x\right)||p\left(y\right)\right)\right)$$

where *p*(*y|x*) represents the conditional class distribution given an image *x*, while *p*(*y*) represents the marginal class distribution. The *KL* divergence is used to quantify the difference between these two distributions. The expected value (*E*) is computed over a set of generated images. Another commonly used metric is the Fréchet Inception Distance (*FID*), which assesses the similarity between the feature representations of real and generated images. The *FID* metric takes into account both the quality and diversity of the generated images. The calculation equation for the *FID* is as follows:

(2) $$FID=||\mu_{r}-\mu_{g}\;{\left|\right|}^{2}+Tr\;(\Sigma r+\Sigma g-2\;\sqrt{\left(\Sigma r\Sigma g\right)}\;)\;$$

where *μ_r_* and *μ_g_* represent the mean feature representations of real and generated images, respectively. Σr and Σg represent the covariance matrices of the real and generated image features.

The *PSNR* (peak signal-to-noise ratio) metric is employed to assess the quality of reconstructed or compressed images. It quantifies the ratio between the maximum achievable power of a signal, like an image, and the power of noise that distorts its fidelity. The *PSNR* is calculated using the following formula:

(3) $$PSNR=10\times \log_{10}\left(\frac{\left(L^{2}\right)}{MSE}\right)$$

where *L* represents the maximum pixel value of the image. *MSE* (mean squared error) refers to the average squared difference between the original image and the reconstructed or compressed version. The *SSIM* (structural similarity index) metric evaluates the perceived structural similarity between two images. It considers factors such as luminance, contrast, and structure, taking into account human visual perception. *SSIM* values fall within the range of −1 to 1, where 1 signifies identical images. The calculation of *SSIM* is performed using the following formula:

(4) $$SSIM=\left(l^{\alpha }\right)\times \left(c^{\beta }\right)\times \left(s^{\gamma }\right)$$

where *l* represents the luminance component, *c* represents the contrast component, and *s* represents the structural component. *α*, *β*, and *γ* are weighting parameters that determine the relative importance of each component. Typically, values of *α* = *β* = *γ* = 1 are used. Additionally, the evaluation framework incorporates *PSNR* and *SSIM* metrics at different levels (0.1, 0.25, 0.5, and 0.75) to assess the effectiveness of noise removal from the images.

The *RMSE* (root mean square error) metric quantifies the average magnitude of differences between predicted and ground truth values in regression tasks. It offers a comprehensive measure of prediction error, where lower *RMSE* values indicate higher accuracy. The calculation of *RMSE* is as follows:

*RMSE = sqrt*((*1/N*) *× Σ*(*y_p_* − *y_t_*)*^2^*) (5)

where *N* represents the number of samples. *y_p_* and *y_t_* denote the predicted and ground truth values, respectively.

In our experiment, we thoroughly assessed our proposed framework by employing multiple performance evaluation indicators, such as the *precision*, *recall*, *accuracy* and *Dice score*.

*Precision* quantifies the ratio of accurately predicted positive instances to the total number of predicted positive instances. *Recall* calculates the ratio of correctly predicted positive instances to the total number of actual positive instances.

(6) $$Precision=\frac{{TP}_{i}}{{TP}_{i}+{FP}_{i}}\times 100\%$$

(7) $$Recall=\frac{{TP}_{i}}{{TP}_{i}+{FN}_{i}}\times 100\%$$

where *TP* (true positives) signifies the number of positive instances that were accurately predicted. *TN* (true negatives) indicates the number of negative instances that were accurately predicted. *FP* (false positives) denotes the count of positive instances that were incorrectly predicted. *FN* (false negatives) conveys the count of negative instances that were inaccurately predicted.

Accuracy, on the other hand, is a crucial metric that evaluates the overall correctness of predictions. It determines the percentage of pixels or instances in the segmentation results that are correctly classified. A higher accuracy score indicates a greater level of accuracy in correctly predicting the segmentation labels.

(8) $$Accuracy=\frac{{TP}_{i}+{TN}_{i}}{{TP}_{i}+{TN}_{i}+{FP}_{i}+{FN}_{i}}\times 100\%$$

The *Dice score*, also referred to as the Dice coefficient or F1 score, is a commonly utilized metric in image segmentation tasks.

(9) $$Dice\;Score=\frac{2\times |\;Precision\times Recall\;|}{\left|\;Precision+Recall\;\right|}\times 100\%$$

where: the expression |Precision×Recall| represents the count of pixels that are present in both the predicted and ground truth segmentations. Overall, the integration of the *Dice score*, *accuracy*, *precision*, and *recall* forms a comprehensive evaluation framework, allowing for a thorough assessment of the capabilities and effectiveness of our proposed approach in the domain of image segmentation and classification.

## 4. Results and Discussion

This section tabulates and discusses the various outcomes for each step in the proposed framework. In Section 4.1, the results of comparing various models for removing noise from retinal fundus images are discussed. The comparison is conducted in terms of PSNR, SSIM, and time. In Section 4.2, the results of comparing different models for data imputation are discussed. These results are based on RMSE and PID evaluation metrics. In Section 4.3, the results of data augmentation are indicted by using IS and FID for the comparison of the utilized models. In Section 4.4, the results of the retinal blood vessel segmentation are presented. The Dice score, accuracy, precision, recall, and time per epoch are used as evaluation metrics.

### 4.1. Results of Removing Noise Layer

**Figure 5:**
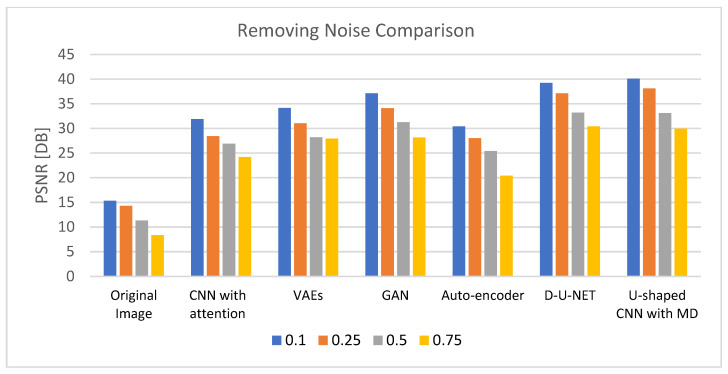
PSNR comparison chart for removing noise from generated images.

Table 3 demonstrates the results of removing noise using different DL models after 200 epochs with a learning rate of 0.0001 and using an Admax optimizer. The comparison is based on four noise levels (0.1, 0.25, 0.5, and 0.75). The outcomes validate the U-shaped CNN with the MD model’s effectiveness in eliminating noise at various degrees of noise when compared to other DL models. The results of the comparison for reducing noise from the retinal images are shown in Figure 5.

### 4.2. Results of Data Imputation Layer

**Figure 6:**
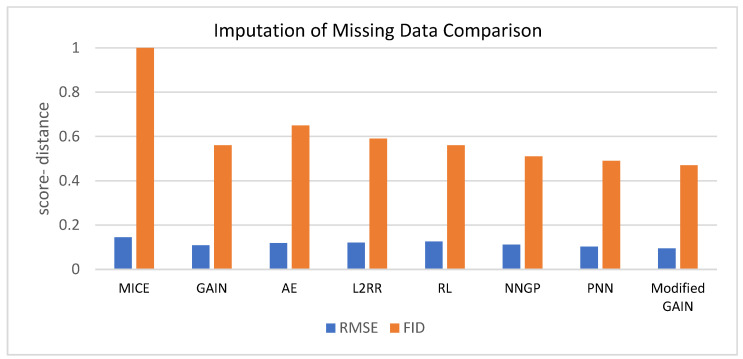
Comparison among various models for data imputation.

Table 4 shows the performance evaluation for the MICE, GAIN, AE, L2RR, RL, NNGP, PNN, and modified GAIN based on RMSE and FID. The findings indicate that the modified GAIN demonstrates superior efficiency when compared to other models for smaller values of RMSE and FID. Figure 6 represents the same data.

### 4.3. Results of Data Augmentation Layer

This section shows the results of augmenting the DRIVE dataset using the LDM and the other architectures of GANs after 200 epochs based on the Adamx optimizer. Table 5 shows the parameters of different architectures for augmenting the Drive dataset. The comparison between the LDM and the various GAN architectures, such as the deep convolutional GAN (DCGAN), vanilla GANs [64,65,66], Wasserstein GAN [67], AGGrGAN [68], and IGAN [69] is shown in Table 6. The results show the efficiency of the LDM in augmentation when compared with different types of GANs during the smaller value of FID and the larger value of IS.

After the data augmentation process, the number of images in the training dataset significantly increased. Prior to augmentation, the training dataset consisted of the original 40 images. However, after incorporating the augmentation techniques, the final training dataset expanded to include a total of 140 images. This augmentation process allowed us to create a more comprehensive and diverse training set, facilitating better generalization and improving the performance of our data imputation algorithm.

### 4.4. Results of Segmentation Stage

This section shows the retinal blood vessel segmentation for retinal images before and after the multi-layer preprocessing stage. 

The final training dataset for U-net consists of a total of N = 140 images, where N represents the number of augmented images generated from the original DRIVE dataset and the original images after the augmentation step. The paper divides the N images into 80% for training and 20% for testing. This augmented dataset provides a richer representation of variations in retinal images, enabling the U-Net model to learn robust features and improve its performance in diabetic retinopathy detection.

**Figure 7:**
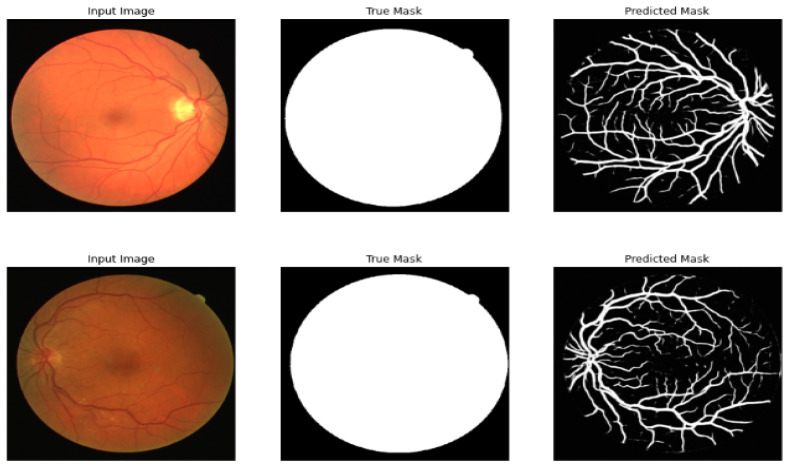
Original image, true mask, and predicted mask using the proposed framework.

Table 7 compares the different models of segmentation before the multi-layer preprocessing stage, and Table 8 shows the results after the multi-layer preprocessing stage. Figure 7 shows the result of segmenting the retinal image after the multi-layer preprocessing stage.

## 5. Statistical Analysis

The statistical analysis of the research presented in this paper focuses on the evaluation of the proposed framework for retinal blood vessel segmentation. The research contributes to the field of medical image analysis, particularly in the context of ophthalmology. The following statistical findings and analysis provide insights into the framework’s performance and its potential applications:

### 5.1. Performance Metrics for Segmentation

- Dice score: the framework achieved an impressive Dice score of 95.32. This metric is a widely used measure in image segmentation, indicating the extent of overlap between the predicted and ground-truth segmentations. A score close to 100 signifies high accuracy in segmenting retinal blood vessels.
- Accuracy: the reported accuracy of 93.56 is another essential metric that measures the proportion of correctly segmented pixels. High accuracy indicates the model’s ability to correctly classify pixels as either blood vessels or background.
- Precision: the precision of 95.68 highlights the framework’s capability to minimize false positives. It signifies the accuracy of positive predictions, reducing the chances of misclassifying non-blood vessel pixels as blood vessels.
- Recall: a recall of 95.45 underscores the model’s effectiveness in identifying true positive cases, minimizing false negatives. It ensures that a significant portion of actual blood vessels is successfully detected.

### 5.2. Noise Reduction Effectiveness

The framework efficiently removes noise from retinal images, as evidenced by the evaluation of the peak signal-to-noise ratio (PSNR) and structural similarity index (SSIM) for varying noise levels (0.1, 0.25, 0.5, and 0.75). These metrics quantify the improvement in image quality after noise reduction, indicating the framework’s ability to enhance image clarity and detail.

### 5.3. Data Augmentation Impact

The latent diffusion model (LDM) used for data augmentation achieved an inception score of 13.6 and a Fréchet Inception Distance (FID) of 46.2 during the augmentation step. These metrics are associated with the quality and diversity of augmented data. A higher inception score suggests that the augmented data closely resemble the original dataset, while a lower FID indicates that the augmented data are similar to the training dataset. These results emphasize the effectiveness of the LDM in generating high-quality additional data for training.

### 5.4. Versatility and Adaptability

The research highlights the versatility of the framework in addressing various challenges such as noisy images, limited datasets, and missing data. While the framework excels in these aspects, it acknowledges limitations in dealing with super-resolution images and generating high-resolution images during augmentation. The framework’s adaptability to real-world scenarios is supported by its comprehensive multi-layer preprocessing approach.

## 6. Conclusions and Future Work

Segmentation of blood vessels is one of the most crucial tasks for many clinicians. This paper provided a new framework for segmenting vessels to detect many diseases. The framework’s two-stage approach, encompassing multi-layer preprocessing and segmentation using a U-Net with a multi-residual attention block, delivers several noteworthy contributions. Firstly, it pioneers the simultaneous use of multi-layer preprocessing with three layers, addressing noise removal, missing data imputation, and dataset augmentation, providing a comprehensive solution to the complexities of retinal image data. Secondly, the framework substantially enhances segmentation performance, demonstrating impressive accuracy and precision. The experiments show that the framework is effective at segmenting retinal blood vessels. It achieved Dice scores of 95.32, accuracy of 93.56, precision of 95.68, and recall of 95.45. Furthermore, it exhibits versatility in tackling challenges such as noisy images, limited datasets, and missing data, all of which are effectively addressed. The U-Net with a multi-residual attention block (MRA-UNet) is used to segment the retinal images after they have been preprocessed and noise has been removed. The experiments also prove the efficiency of the segmentation model. The results also show improvements in different architectures of the U-Net after the multi-layer preprocessing. Although the framework presented good results in all sections, it still has some limitations in dealing with super-resolution images and generating high-resolution images in the augmentation step. In the future, we will use the super-resolution diffusion model to generate new samples to improve the accuracy of the segmentation process, and we will use the diffusion model to remove noise.

## Figures and Tables

**Table 1:** Segmentation of retinal blood vessels based on different architectures of the U-Net.

Ref	DL Model	Task	Advantages	Disadvantages
[37]	Improved U-Net	Segmentation and detection	Accuracy	Cannot deal with noisy images Cannot complete the training procedure with a restricted quantity of photos
[39]	U-Net++	Segmentation	Accuracy	Cannot deal with noisy images Cannot complete the training procedure with a restricted quantity of photos
[43]	SA-UNet	Segmentation	Network substitutes structured dropout convolutional blocks for the original U-Net.	Cannot deal with noisy images Cannot complete the training procedure with a restricted quantity of photos Accuracy
[44]	DEU-Net	Segmentation	Accuracy	Cannot deal with noisy images Cannot complete the training procedure with a restricted quantity of photos
[45]	Vessel-Net	Segmentation	Accuracy and preprocessing step	Cannot complete the training procedure with a restricted quantity of photos

**Table 2:** Hardware and software specification for the experimental results.

Device	Description
Processors	Intel(R) Core(TM) i7-10750H CPU @ 2.60 GHz
Random Access Memory	64.0 GB
Graphical Processing Unit	NVIDIA GeForce RTX 3050Ti
Space	2 TB
Programming language	Python

**Table 3:** Performance evaluation of removing noise for various models.

Method	PSNR			SSIM					Time
	0.1	0.25	0.5	0.75	0.1	0.25	0.5	0.75	
Original Image	15.31	14.31	11.34	8.34	67.31%	60.30%	50.02%	39.01%	
CNN with attention	31.89	28.45	26.89	24.19	88.49%	81.26%	78.12%	73.15%	24.98
VAEs	34.15	31.06	28.19	27.94	91.11%	86.14%	81.69%	78.16%	24.98
GAN	37.11	34.11	31.28	28.17	91.71%	89.13%	86.49%	82.09%	24.46
Auto-encoder	30.43	28.01	25.43	20.43	82.31%	79.42%	75.21%	70.31%	24.04
D-U-NET	39.23	37.14	33.21	30.42	94.41%	91.09%	88.01%	83.21%	23.13
U-shaped CNN with MD	40.09	38.11	33.10	29.97	94.63%	92.00%	89.23%	84.65%	24.03

**Table 4:** Performance evaluation of data imputation techniques.

Model	RMSE	FID
MICE	0.145	1
GAIN	0.109	0.56
AE	0.119	0.65
L2RR	0.121	0.59
RL	0.126	0.56
NNGP	0.112	0.51
PNN	0.103	0.49
Modified GAIN	0.0945	0.47

**Table 5:** Proposed model parameters.

Model	Minimum Batch Size	Epochs Number	Rate of Discriminator-Generator Learning	Rate of Generator Learning
MGAN	128	200	0.0001–0.0002	Adam
DCGAN	128	200	0.0001–0.0002	Adam
Vanilla GAN	64	200	0.0001–0.0002	Adam
Wasserstein GAN	128	200	0.0001–0.0002	Adam with gradient penalty
AGGrGAN	64	200	0.0001–0.0002	Adam
IGAN	64	200	0.0001–0.0002	Adam

**Table 6:** Performance evaluation of data augmentation models.

Model	IS	FID
LDM	13.6	43.7
MGAN	12.6	47.7
DCGAN	11.7	47.9
Vanilla GAN	10.23	49.2
Wasserstein GAN	12.45	45.32
MG-CWGAN	10.36	44.29
AGGrGAN	11.46	45.23
IGAN	11.78	45.69

**Table 7:** Segmentation-based comparison of different models before multi-layer preprocessing stage.

Model	Dice Score	Accuracy	Precision	Recall	Time per Epoch
Attention gate U-Net	91.27	91.68	91.11	90.89	23.1
U-Net	87.36	88.01	88.69	88.46	24.6
U-Net++	91.53	91.59	91.67	91.36	25.3
RA-UNet++	92.01	92.58	92.83	92.77	24.6
SA-UNet	92.68	92.67	92.67	92.09	23.1
DEU-Net	91.93	91.55	92.35	92.23	23.6
UNet 3+	92.12	91.78	92.68	92.11	24.1
MRA-UNet	93.68	93.25	93.16	93.57	23.5

**Table 8:** Segmentation-based comparison of different models after multi-layer preprocessing stage.

Model	Dice Score	Accuracy	Precision	Recall
Attention gate U-Net	92.54	92.37	92.56	92.65
U-Net	90.16	90.11	90.29	90.55
U-Net++	92.52	92.47	92.71	92.24
RA-UNet++	93.01	93.37	93.63	93.57
SA-UNet	93.48	93.58	93.88	93.19
DEU-Net	93.25	93.44	93.28	93.28
UNet 3+	93.91	93.67	93.48	93.15
MRA-UNet	95.32	93.56	95.68	95.45

## Data Availability

Furnished on request.
